# Walking and finger tapping can be done with independent rhythms

**DOI:** 10.1038/s41598-019-43824-0

**Published:** 2019-05-20

**Authors:** Weihuang Qi, Tsuyoshi Nakajima, Masanori Sakamoto, Kouki Kato, Yasuo Kawakami, Kazuyuki Kanosue

**Affiliations:** 1 Graduate School of Sport Sciences, Waseda University, Saitama, Japan; 2 Department of Integrative Physiology, Kyorin University School of Medicine, Tokyo, Japan; 3 Faculty of Education, Department of Physical Education, Kumamoto University, Kumamoto, Japan; 4 Faculty of Sport Sciences, Waseda University, Saitama, Japan

**Keywords:** Cognitive control, Central pattern generators, Spinal cord

## Abstract

Rhythmic movements occur in many aspects of daily life. Examples include clapping the hands and walking. The production of two independent rhythms with multiple limbs is considered to be extremely difficult. In the present study we evaluated whether two different, independent rhythms that involved finger tapping and walking could be produced. In Experiment I, twenty subjects that had no experience of musical instrument training performed rhythmic finger tapping with the right index finger and one of four different lower limb movements; (1) self-paced walking, (2) given-paced walking, (3) alternative bilateral heel tapping from a sitting position, and (4) unilateral heel tapping with the leg ipsilateral to the tapping finger from a sitting position. The target intervals of finger tapping and heel strikes for walking step/heel tapping were set at 375 ms and 600 ms, respectively. The even distribution of relative phases between instantaneous finger tapping and heel strike was taken as the criteria of independency for the two rhythms. In the self-paced walking and given-paced walking tasks, 16 out of 20 subjects successfully performed finger tapping and walking with independent rhythms without any special practice. On the other hand, in the bipedal heels striking and unipedal heel striking tasks 19 subjects failed to perform the two movements independently, falling into interrelated rhythms with the ratio mostly being 2:1. In Experiment II, a similar independency of finger tapping and walking at a given pace was observed for heel strike intervals of 400, 600, and 800 ms, as well as at the constant 375 ms for finger tapping. These results suggest that finger tapping and walking are controlled by separate neural control mechanisms, presumably with a supra-spinal locus for finger tapping, and a spinal location for walking.

## Introduction

Rhythmic movements occur in many aspects of daily life. Examples include clapping the hands and walking. Rhythmic movements do not always occur alone, and in complex situations, like playing a drum set, two or more rhythms are performed simultaneously. Rhythmic movements such as finger tapping are related to neural mechanisms located in supra-spinal structures^1^. The basal ganglia and cerebellum play important roles in the process of perceiving or producing rhythmic time intervals, especially those of short duration (up to seconds)^2,3^. Because of this, repetitive finger tapping has been widely utilized as an experimental model to study temporal perception and motor timing^4^. Simultaneously producing two kinds of rhythms with a different tempo (polyrhythm) is extremely difficult, and the only way humans can produce a polyrhythm is considered to integrate two rhythms into one complex rhythm rather than by performing two rhythms in parallel^5–9^. For example, although drummers can seemingly beat a drum set polyrhythmically with the arms and legs (such as a 3 beat with arms and a 4 beat with legs), they do not actually generate independent rhythms in parallel but rather integrate those two rhythms into a single complex rhythm sequence involving arm and leg movements^10^. However, whether or not independent polyrhythmic movements with multiple limbs are truly impossible in any situation remains unclear.

Another model system used to study rhythmic movements involves locomotion. While the polyrhythm studies largely focused on bimanual movements, lower limb movements have been mainly analyzed in locomotion studies. The above distinction likely occurred because locomotion is accomplished with the lower limbs in humans. For quadrupedal animals, locomotor movements involving alternative bilateral activation of muscles can be produced only with neural networks in the spinal cord^11–13^. Thus, cats with a spinal cord transection and severed dorsal roots can still produce rhythmic muscle activation that involves alternative ankle flexion and extension^14,15^. A rhythm generating mechanism is located on each side of the lumbar and cervical spinal cord, and the mechanisms on each side are connected via commissural fibers which allow the creation of alternative activation in the muscles^16–19^. Indirect evidence of a similar mechanism in humans has also been presented^13,20–25^. When the lumbar spinal cord of spinal cord injury patients is stimulated with non-patterned electrical stimulation, step-like EMG activity and locomotor synergies can be observed^26^. Overall activity of the spinal mechanisms is modulated by signals from supra-spinal structures. These signals allow for such things as intentionally changing the pace of walking or running^27,28^.

Sakamoto *et al*.^29^ performed an interesting experiment in which subjects performed arm and leg cycling simultaneously at their preferred cadence. Under this condition, the subjects always effected arm and leg cycling with the same cadence. Next, they were asked to change the cadence of either the arm or leg cycling. In this situation, the cadence of arm cycling was significantly influenced by the change in leg cadence, while the cadence of leg cycling was not influenced by the change in arm cadence. Although the authors did not comment on this possibility, their results suggest the possibility that the upper and lower limbs could be moved with independent rhythms.

It is commonly assumed that the timing of two simultaneously-moving limbs is not independent, and is subsumed under a single, integrated, and hierarchical temporal structure^6,30–32^. However, performing two independent rhythms might be possible if one rhythm involves finger tapping (controlled by supra-spinal structures), and the other rhythm involves walking. This latter movement entails a lower-limb movement that is mainly controlled by circuits in the spinal cord.

The aim of the present study was to test this possibility. Subjects performed two different rhythms which involved finger tapping and walking/heel tapping. The two rhythms were set so that they did not have a simple integer ratio, such as 1:2 or 2:3. This made it difficult for the subjects to perform the ratio by developing one complex rhythm^7,33,34^. The lower limb movements were: (1) self-paced walking; (2) given-paced walking; (3) alternative bilateral heel tapping from a sitting position; and (4) unilateral heel tapping with the leg ipsilateral leg to the tapping finger from a sitting position. During walking the locomotor system is subjected to repetitive impacts involving heel strikes^35^. We utilized the bipedal heels striking task, because it is similar to walking in that it is a bilateral and alternative movement. The unipedal heel striking task is a frequently studied polyrhythmic movement that has been shown to be difficult to perform without specific training^31,34,36,37^. Our null-hypothesis was that finger tapping and lower-limb movements could not be done independently for any of the four tasks.

## Results

### Experiment I

**Figure 1:**
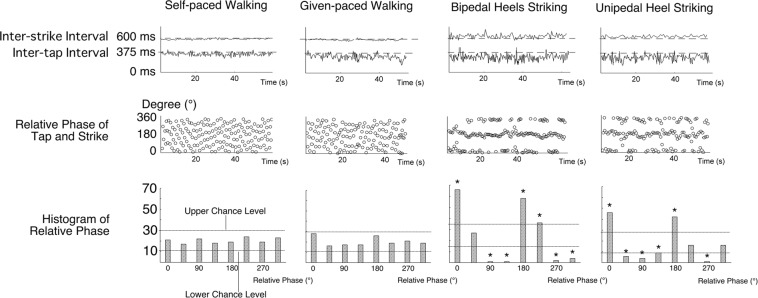
Representative examples of responses for four tasks (self-paced walking, given-paced walking, bipedal heels striking, and unipedal heel striking) in one subject. The top graphs: Temporal changes in inter-strike and inter-tap intervals. Dashed lines indicate the target time intervals, 375 ms for finger taps and 600 ms for heel strikes. The middle graphs: Instantaneous relative phases of finger tapping and heel strikes. The bottom histogram: Number of the relative phase that occurred in 45° bins centered from 0° to 315°. Two lines in each histogram indicate the upper and lower limits of the chance level. ^*^Indicates a value outside the limits (P < 0.05).

An example of finger tapping and heel striking in one typical subject for the four kinds of dual motor tasks is shown in Fig. 1. All task intervals of heel strikes (denoted as the inter-strike interval in the following) were close to the target (600 ms, dashed lines) but they fluctuated more in the bipedal heels striking and unipedal heel tasks. Inter-tap intervals tended to deviate from, and became lower than, the target (375 ms, dashed line). This was particularly evident for the bipedal heels striking and unipedal heel striking tasks.

**Figure 2:**
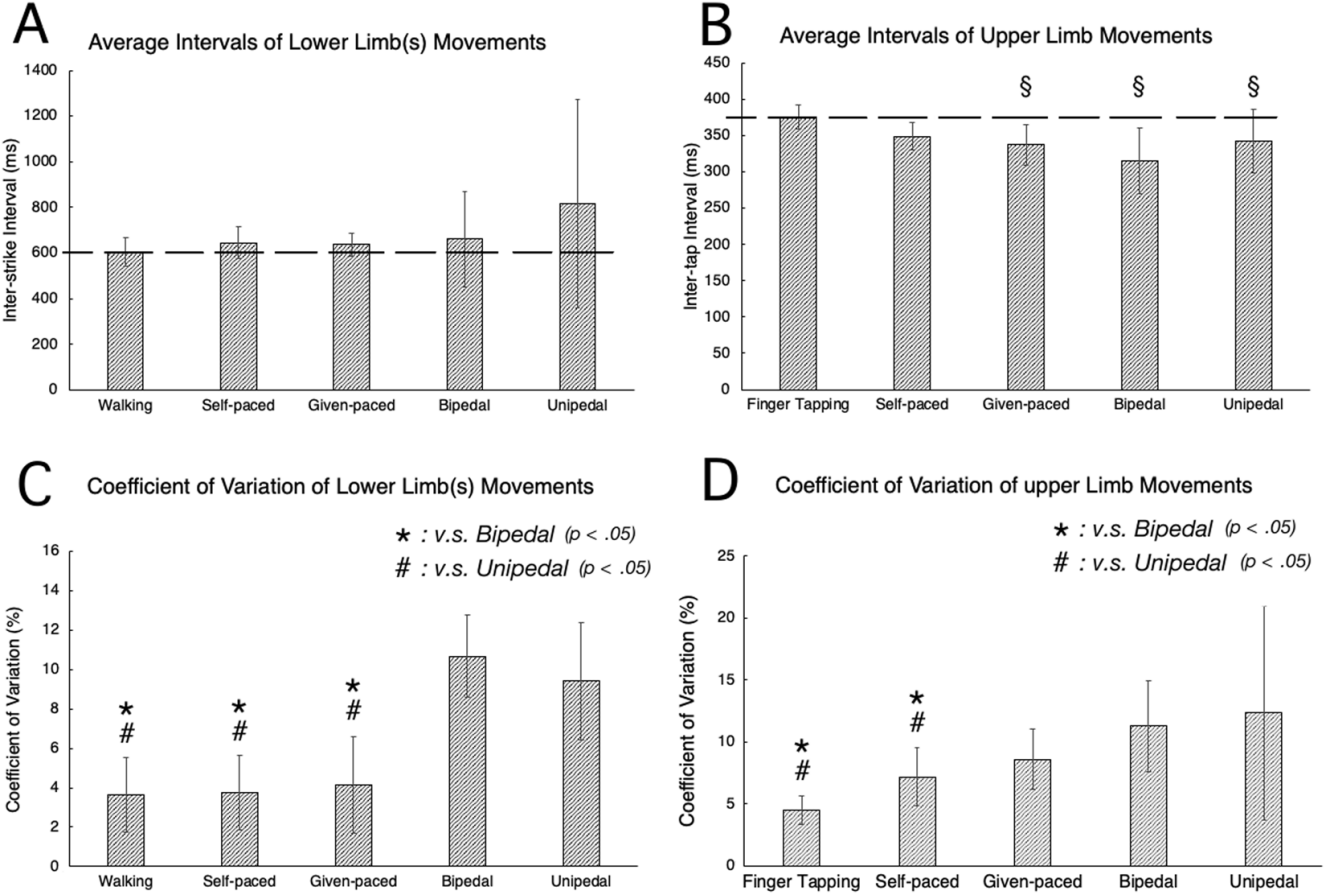
(**A)** Average time intervals for the heel strike. The broken line indicates the target time interval, 600 ms. (**B**) Average time intervals for finger tapping. The broken line indicates the target time interval, 375 ms. (**C**) Coefficient of variation for the heel strike. (**D**) Coefficient of variation for finger tapping. Bars are s.d. ^§^ indicates a significant difference from the target interval. * and ^#^ indicate significant differences from bipedal and unipedal heel striking tasks, respectively (P < 0.05).

Figure 2 shows the properties of each of the upper and lower limb movements. For the averaged interval of heel strikes, no task, including the single task of walking, showed a significant difference from the target interval (600 ms, dashed line) (F[5, 115] = 2.286, *p* = 0.07) (Fig. 2A). On the other hand, for the averaged intervals of finger tapings, during given paced walking, bipedal heel striking and unipedal heel striking tasks showed a significant difference from the target interval (375 ms, dashed line) (F[5, 115] = 33.817, *p* = 0.00 for bipedal heel striking and F[5, 115] = 33.056, *p* = 0.00 for unipedal heel striking). The single task of finger tapping and the self-paced walking task showed no significant difference from the target interval (Fig. 2B). For the coefficient of variation (CV) of the inter-strike interval (Fig. 2C), a significant interaction was found across the tasks (F[4, 76] = 33.780, *p* = 0.00) and CV of the walking, self-paced walking and given-paced walking tasks were significantly lower than those of the bipedal heels striking tasks (*p* = 0.00 with both self-paced walking task and given-paced walking task) and unipedal heel tasks and unipedal heel striking tasks (*p* = 0.00 with both self-paced walking task and given-paced walking tasks). Likewise, an interaction of CV for the inter-tap intervals (Fig. 2D) was also found across the tasks (F[4, 66] = 7.172, *p* = 0.00) and CVs of the finger tapping task, self-paced walking were significantly lower than those of the bipedal heels striking (*p* = 0.00 with finger tapping task and *p* = 0.04 with self-paced walking task) and unipedal heel striking tasks (*p* = 0.00 with finger tapping task and *p* = 0.02 with self-paced tapping task). There was no significant difference from given-paced walking task to other tasks.

The interaction between finger tapping and heel strikes as well as their independency were analyzed by comparing the relative phases of the two movements. This analysis of each task utilized only the data of subjects in which the CVs of either finger tapping or heel strikes was lower than the threshold of exclusion (15%). With this criterion 1, 1, 8, and 12 subjects in the self-paced walking, given-paced walking, bipedal heels striking and unipedal heel striking tasks, respectively, were excluded from the subsequent analysis of independency. Thus, the number of subjects left for further analysis of independency were: 19 in the self-paced walking task, 19 in the given-paced walking task, 12 in the bipedal heels striking task, and 8 in the unipedal heel striking task.

In the example seen in Fig. 1, different patterns in the relative phase distribution can be observed between the self-paced walking/given-paced walking tasks and bipedal heels/unipedal heel striking tasks (the scatter plots of Fig. 1). While the instantaneous relative phases scatter evenly across the whole range from 0° to 360° for the self-paced walking/given-paced walking tasks, they accumulate around 0°/360° and 180° for the bipedal heels/unipedal heel tasks. The same tendency can be quantitatively observed in the histograms (the bottom of Fig. 1). For the self-paced walking and given-paced walking tasks, the values of all the 45° bins were within the upper and lower limits of the chance level. On the other hand, for the bipedal heels striking and unipedal heel striking tasks, the values are significantly above the upper limit (~0° and 180°), or significantly below the lower limit (~90° and 270°). This indicates that finger tapping and heel tapping dropped into a 2:1 rhythm; that is, the subject failed to perform independent movements of the finger and legs. The number of subjects whose data showed histograms with no bins outside (higher or lower than) the chance levels were 16/19 in the self-paced walking task, 16/19 in the given-paced walking task, 1/12 in the bipedal heel striking task, and 1/8 in the unipedal heel striking task (denominators indicate the number of subjects analyzed).

**Figure 3:**
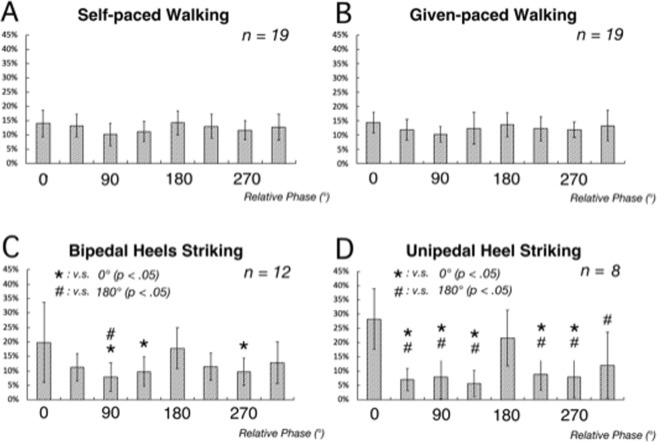
Normalized distribution of the relative phase of the group of subjects after exclusion of those subjects whose performance stability (CV) of finger tapping or walking was greater than 15%. (**A**) Self-paced walking task. (**B**) Given-paced walking task. (**C**) Bipedal heel striking task. (**D**) Unipedal heel striking task. Bars are s.d. * and ^#^ indicate significant differences from the bars centering on 0° and 180°, respectively.

Figure 3 shows the results for group analysis of relative phase in the four kinds of dual tasks (the histograms of distribution of relative phase, normalized by making the total of values in all bins as 100%).The bipedal heel striking task rejected the homoscedasticity test (F[7, 96] = 4.22, *p* = 0.00) so the Kruskal-Wallis test was utilized. This test indicated a significant difference (H = 21.26, *df* = 7, *p* = 0.00) among the values (Fig. 3C). In the self-paced walking (Fig. 3A) and given-paced walking tasks (Fig. 3B), no significance was obtained in the post hoc analysis among all eight bins. In the bipedal heel striking task (Fig. 3C), the value in the 45° bin centering on 0° was significantly higher than those in the bins centering on 90° (*p* = 0.00), 135° (*p* = 0.02), and 270° (*p* = 0.02). Likewise, the value in the bin centering on 180° was significantly higher than that in the bin centering on 90° (*p* = 0.03). In the unipedal heel striking task (Fig. 3D), the value in the bin centering on 0° was significantly higher than those in all the bins (*p* = 0.00) except that in the bin centering on 180°. The value in the bin centering on 180° was also significantly higher than the other bars (*p* < 0.01) excepting the bars centering on 0° and 315°.

### Experiment II

**Figure 4:**
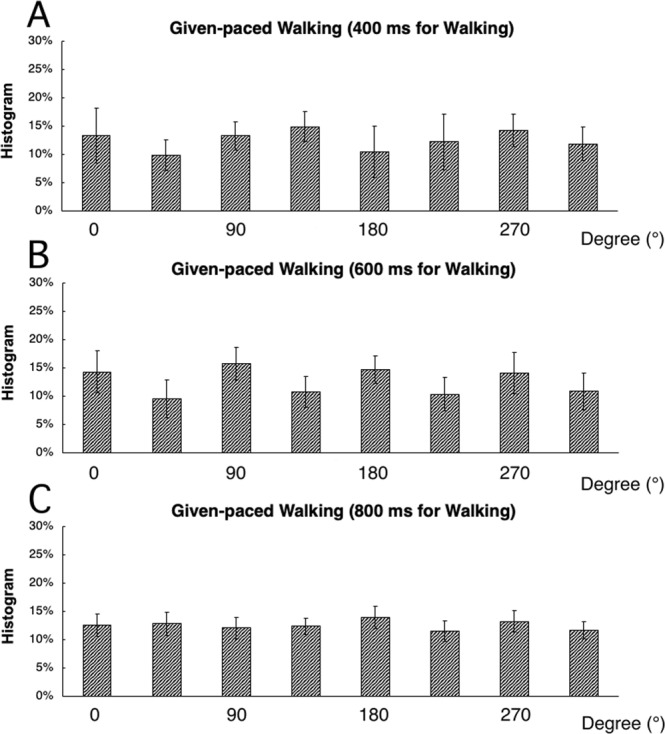
Normalized distribution of the relative phase of the given-paced walking task for walking intervals of 400 ms (**A**), 600 ms (**B**) and 800 ms (**C**). Bars are s.d.

In Experiment I, we found that an even distribution of relative phases of finger tapping and heel strike could be produced with the self-paced walking and given-paced walking tasks. This was contrary to the null-hypothesis and indicates the possibility of independent movement for the upper and lower limbs. However, this independence might be the result of the special combination of intervals of finger tapping (375 ms) and heel strike (600 ms). Therefore, we performed an additional experiment with 15 subjects (not necessarily those involved in the Experiment 1) for the given-paced walking task in which two other interval lengths (400 ms and 800 ms) were adopted for heel striking, as well as the original 600 ms. A constant interval was used for finger tapping (375 ms). Utilizing the criteria of 15% CV of either interval length of finger tapping or walking resulted in no subject being excluded. Figure 4 indicates that there was no significant difference among 45° bins of relative phase not only in the condition of 600 ms interval for walking (Fig. 4B), similar to the result of Experiment I, but also in the other two conditions of 400 ms (Fig. 4A) and 800 ms (Fig. 4C) (F[7, 56] = 0.00, *p* = 1.00 for 400 ms walking, F[7, 56] = 0.02, *p* = 1.00 for 600 ms walking, and F[7, 56] = 0.00, *p* = 1.00 for 800 ms walking).

## Discussion

While rhythms of finger tapping and walking are both very stable when they were done alone, they became unstable in some subjects in all dual task conditions. Therefore, before testing whether hand and leg rhythmic movements could be produced independently, we analyzed the stability of each movement. Great instability (CV > 15%) was observed in 1 subject in the self-paced walking, 1 in the given-paced walking task, 8 in the bipedal heels striking task, and 12 in the unipedal heel striking task. As a group data, both leg and finger movements showed bigger CVs in the bipedal heels striking or unipedal heel striking tasks than in the self-paced walking and given-paced walking tasks (Fig. 2C,D). This indicates that simultaneous movements of finger and legs are more difficult in the bipedal heel striking and unipedal heel striking tasks (moving legs from the sitting position) than in the self-paced walking and given-paced walking tasks. And those subjects with CVs greater than the criterion were excluded from the following analysis of independence. In the rest of subjects, the rhythm of finger and legs were relatively stable and different from each other (Fig. 2A,B).

However, a difference in rhythm alone does not necessarily indicate/guarantee that the two movements were “independent”. Therefore, the tendency to be independent for the two movements was evaluated by examining the distribution of relative phases of the two movements. We made a histogram of the relative phase by dividing the whole range (0° to 360°) into 8 bins of 45° as shown in the bottom portion of Fig. 1. The criterion for independence was an even distribution of the bins. That is, if all the values were within the upper and lower limits of the chance level, the finger tapping and leg movements would be considered as independent. Sixteen out of 20 subjects satisfied this criterion for the self-paced walking and given-paced walking tasks, while only one subject did in the bipedal heel striking and unipedal heel tasks.

In order to conclude that independent rhythms of finger tapping and walking have occurred, several issues have to be considered. First, in Experiment 1, we only tested 2 rhythms, which were multiples of the same fundamental period (25 ms) that repeats every 3 seconds (8 taps or 5 heel strikes), or a 5 over 4 polyrhythm (with double time in the 4). Although we had considered that the combination of 8:5 (or 4:5) rhythms is complex enough not to easily produce a polyrhythm, producing this polyrhythm might be straightforward for some subjects. However, we recruited only subjects who had no experience of playing musical instruments. And if the success of independent rhythms had been due to that kind of special ability or training, the independence could have been observed not only in the tasks of walking but also in the bipedal and unipedal heel striking, and practice over a relatively long period should have been needed. Therefore, the independent rhythms observed in the self-paced walking and given-paced walking are likely produced by a different mechanism from the subjects’ high level of ability in generating polyrhythms. Furthermore, we performed additional experiments with different rhythm ratios (375 ms vs 400 ms or 800 ms, 15:16 or 15: 32) (Experiment II). Even in these very complex ratios, the results were basically the same as those observed with the original rhythm ratio (375 ms vs 600 ms, 5:8). This strongly indicates that the polyrhythms observed in the self-paced walking and given-paced walking tasks are not due to an unusually high ability level of the subjects.

Second, it might be the case that the rhythm of walking was governed by the plant dynamics of musculoskeletal system of the lower limbs, and the inter-strike period of 600 ms by chance matched the oscillating frequency of the “walking plant” of the subjects. This would allow the subjects to concentrate on the finger tapping. This possibility was also rejected by the results of the Experiment II, in which two other inter-strike periods (400 ms and 800 ms) were tested. Independent movements could be observed for these other two inter-strike intervals as well as for the original 600 ms interval. Therefore, it could not be the case that the independency of upper and lower limb movements occurred by chance due to special plant dynamics.

Finally, Mechsner^38^ proposed that bimanual coordination with different rhythms for both arms was possible by accomplishing one’s “perceptual goal”. This was demonstrated by instructing subjects to circle two visible flags by way of two cranks without visual information from the moving arms. The flags moved in accordance with the movement of the cranks, but each crank had a different gear ratio so that a 4:3 frequency ratio of the cranks resulted in an iso-frequency in the flags. Bimanual movements with a non-harmonic frequency relationship (i.e., 4:3), are considered to be almost impossible to perform for naïve subjects. However, such movements could be performed under the visual feedback of flags with the simplest frequency relationship (i.e., 1:1). The subjects in Mechsner’s experiment paid no attention to the rhythms of both hands and rather concentrated on other cues (movement of the flags). However, subjects in the current study focused on rhythms for the upper and lower limb movements themselves, at least when they started each movement. In regard to this point there is no difference between the walking tasks (self-paced walking and given-paced walking) and the stepping tasks (bipedal heel striking and unipedal heel striking), although the performance was drastically different between the two task groups. Thus, it is improbable that the success of independent rhythms in the self-paced walking and given-paced walking tasks was due to any special condition of perception.

Thus, it is quite likely that the majority of subjects, who showed an even relative phase distribution in the histogram, could perform finger tapping and walking (the self-paced walking and given-paced walking tasks) with independent rhythms. This was accomplished with no special practice.

A different pattern of the relative phase histogram distribution was obtained with the remaining four subjects for the self-paced walking and given-paced walking tasks and in most subjects for the bipedal heels striking and unipedal heel striking tasks. In these cases, there was a tendency for relative phase accumulation around specific angles. This signifies an interaction between finger tapping and walking/heel tapping. Furthermore, the significantly larger values of relative phase at ~0° and 180° indicates that the two movements fell into a synchronized 2:1 pattern: that is, they were not independent. Thus, 4 of 20 subjects in the self-paced walking and given-paced walking tasks and 19 of 20 subjects in the bipedal heels striking and unipedal heel striking tasks failed to produce independent rhythms.

In 16 of 20 subjects, independent rhythms of finger tapping and walking could be produced for both the self-paced walking and given-paced walking tasks. As noted in the introduction, walking and finger movement are considered to be controlled by mechanisms in distinct portions of the nervous system^39,40^. A functional network at the spinal level contributes to the control of human locomotor activities^12,24,41^, while rhythmic finger tapping is orchestrated in supra-spinal structures including the SMA, cerebellum and basal ganglia^42,43^. Thus, we can infer that independent rhythms could be performed in the self-paced walking and given-paced walking tasks because the rhythms of finger tapping and walking were generated by very different neural structures. However, a question remains concerning the given-paced walking task. The difficulty in performing rhythmic dual motor tasks is considered to come from the conflict involved in the sharing of a common motor control system^32^. Since the pace of walking was intentionally set for the given-paced walking task, supra-spinal structures would inevitably be involved in the process, at least to the extent of giving signals concerning the pace of walking to the spinal output mechanism. Consequently, walking at a given pace might share the same motor system with rhythmic finger tapping and thus cause a constraint. Interestingly, in the given-paced walking task, independent rhythms for finger tapping and walking were produced just as accurately as those of the self-paced walking task. There are two possible reasons for this. First, the supra-spinal structures controlling the pace of walking are different from those that control finger tapping. This allows an accurate, independent production of the two movements. Second, the pace of walking might be maintained in the spinal cord, and once the spinal mechanism established the pace, it could produce the walking rhythm without the further strong links with the supra-spinal structures.

For the bipedal heels striking and unipedal heel striking tasks, most subjects (19 of 20) failed to perform finger tapping and leg movements independently. In the case of the unipedal heel striking task, this result agrees with the results of previous studies which found that that two rhythms could not be generated without intense practice, except when the rhythms were in a simple integer ratio such as 2:1^44,45^. In the case of a 2:1 rhythm, finger tapping would occur simultaneously with heel tapping and also in between taps. This pattern is one of the easiest forms of multi-limb/multi-finger polyrhythmic movement^44,46,47^ and appeared many times in the present study. Although the subjects initially attempted to perform the task with independent movements of the finger and legs, they were unintentionally drawn into the more easily synchronized pattern^48,49^.

In general, difficult coordination of movement tasks require a great deal of practice to master (indicating a large cost of learning)^48,50^. Although there is no clear system for categorizing the level of difficulty, researchers believe that the more complex the ratio of polyrhythm is, the more difficult it is to master^6,31,32,51^. For example, higher-order ratios such as 5:4 and 4:3 are much more difficult to perform than are lower-order ratios such as 2:1 or 1:1^52,53^. The task utilized in the present study required performing simultaneous rhythmic movements with intervals of 375 ms and 600 ms. This can be considered to be a 5:8 polyrhythm, and no training was provided before the experiment. Most subjects successfully finished dual motor tasks in the self-paced walking and given-paced walking conditions without any practice. Therefore, we suggest that locomotor movement and rhythmic finger tapping can be performed without practice, even for higher-order ratio polyrhythms.

Although the results of stability and relative phases did indicate that independent rhythmic movements could be done, the movements were not necessarily as accurate as required. That is, the intervals of finger tapping deviated from the target 375 ms interval to a shorter value, and the intervals of heel tapping also deviated from the targeted 600 ms to a longer value (though not necessarily significant). Such deviations were not observed in the single task of each movement (Fig. 2A,B). The mechanisms involved in producing these interval deviations cannot be identified with the results of the current experiments. Their occurrence could be related to the way in which the two target intervals are set or to the neuronal connections between upper and lower limbs^25,54–57^. This is a limitation of the present study and should be addressed in future studies. However, the independency of finger and leg movements could be assured from the randomness of relative phase of movements and its stability regardless of the interval deviation from the target.

## Conclusion

The present study investigated the possibility of performing independent rhythmic movements without special training. The results showed that 16 out of 20 subjects performed finger tapping and self-paced or given-paced walking with an even distribution of relative phase for the upper and lower limb movements. For the tasks with finger tapping and bipedal heel striking or unipedal heel striking all but one subject showed unstable movements, and most attempts collapsed into simple 2:1 rhythms. Thus, two independent rhythms can be made, with finger tapping for one rhythm and walking for the other rhythm.

## Material and Methods

### Setting

**Figure 5:**
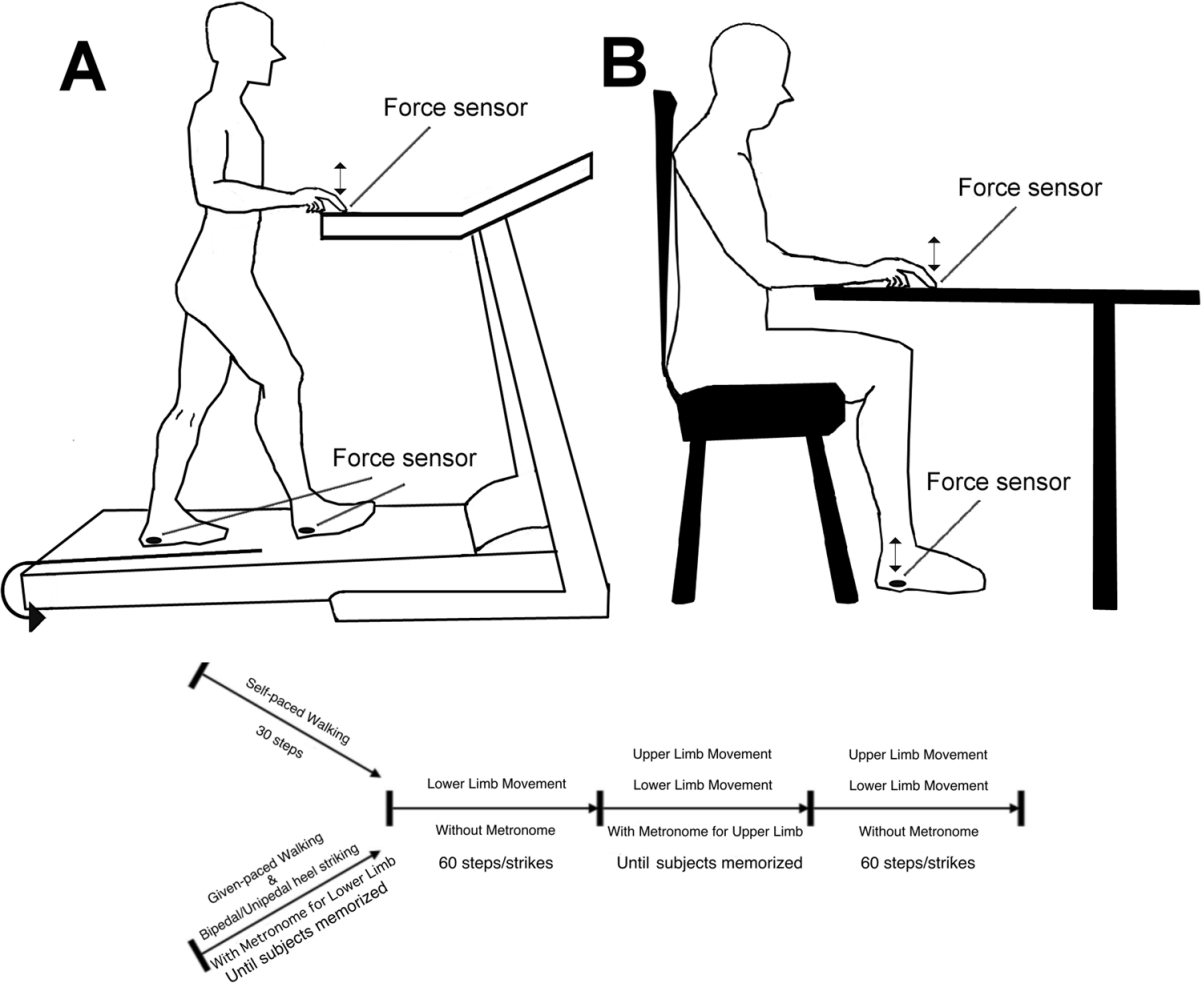
Experimental set up of the self-paced walking task and given-paced walking tasks (**A**) as well as bipedal heels striking and unipedal heel striking tasks (**B**). Force sensors were fixed to the right index finger and under the heels. The experimental protocol is depicted at the bottom.

This study was approved by the Human Research Ethics committee of Waseda University. The experiments were conducted in accordance with the Declaration of Helsinki. Subjects who participated in the experiments were 20–30 years of age, healthy, and right-handed as tested by the Edinburgh handedness inventory^58^. None of them had received training to play musical instruments. All participants received a detailed explanation of the experimental procedures before the experiment, and written informed consent was obtained from all participants. Force sensors (S200 Load Sensor, Biometrics, UK) were fixed on the tip of the right index finger and both heels. In the walking-related tasks, subjects passively walked^59^ on a treadmill (SportsArt 3100 Multi-Mode Treadmill, Germany) and tapped the control panel of the treadmill with the right index finger. For the other tasks, subjects sat at a table upon which they tapped with the right index finger. They were asked to tap the floor with their right heel or both heels alternatively, without having their toes leave the floor (Fig. 5). The signal from the force sensors and metronomes (which supplied target intervals) were fed into a computer through an A/D converter (Powerlab ML880, AD instruments, Australia) at a sampling rate of 1000 Hz.

### Procedures

#### Experiment I

Twenty subjects (11males and 9 females) without musical training performed rhythmic finger tapping with the right index finger and either of four different lower limb movements; the self-paced walking, given-paced walking, bipedal heels striking, and unipedal heel striking tasks. The protocol is depicted on the bottom of Fig. 5. In preparation for the experiments, we recorded the subjects’ preferred walking speed on the treadmill and their step intervals. The mean preferred treadmill speed was 3.1 ± 0.7 km/h and the preferred interval was 593 ± 56 ms. Thus, the target intervals for walking and step/heel tapping were set at 600 ms. The target interval for finger tapping was set at 375 ms, because subjects can easily perform finger tapping at this time interval and the ratio of intervals between the upper and lower limbs (375 ms to 600 ms, 5:8) is not a simple integer ratio. For the self-paced walking and given-paced walking tasks, the treadmill was set at each subject’s preferred speed. For the self-paced walking task, subjects first walked on the treadmill at their preferred step interval for at least 60 steps; this step interval was then taken as the control (basic) value for uncomplicated walking. Then, metronome sound signals (375 ms interval) were presented so the subject could synchronize finger tapping while keeping the walking pace unchanged. After another 60 steps of walking, the metronome was stopped. Then, the subjects were instructed to continue walking and finger tapping without the metronome signal for at least 60 steps. In the given-paced walking task the subjects also first walked on the treadmill, but synchronized their steps with a metronome 600 ms interval signal. The metronome was stopped when the subjects felt they could maintain the tempo. Then they continued walking for a minimum of 60 steps. Subsequently, another metronome signal (375 ms interval) was given, and subjects were asked to synchronize their finger tapping with the metronome while maintaining the same walking pace. When the subjects felt they were maintaining the tempo, the metronome was stopped and the subjects continued finger tapping and walking for a minimum of 60 steps. For the bipedal heels striking and unipedal heel striking tasks, the experimental procedures were the same as for the given-paced walking task, except that the subjects sat. They initiated the task with bilateral or unilateral heel tapping instead of walking. A single task of at least 60 finger taps was separately done as the control (basic value) for uncomplicated finger tapping. In all of the tasks, the subject could see and hear their own performance of both finger tapping and leg movements. This provided both visual and auditory feedback. No training, other than a short period of habituation to the experimental procedure was provided before the experiment. Task order was randomized across subjects. Data acquisition started after the subjects became familiar with all aspects of the tasks.

#### Experiment II

An additional experiment was conducted to test the effects of different lower limb paces. Fifteen subjects (10 males and 5 females, 9 of whom participated in the Experiment 1) were recruited and each subject finished the additional experiment in one day. The given-paced walking task used 3 different walking intervals (400 ms, 600 ms, and 800 ms) and was performed with a random order. The experimental setting and protocol were the same as those of the given-paced walking task in Experiment I.

### Data analysis

For the single control tasks (walking and finger tapping), force data from 60 walking steps and finger taps were used. Likewise, for the self-paced walking, given-paced walking, bipedal heels striking and unipedal heel striking tasks, the data was taken from the period of dual motor tasks without the metronome signal over 60 heel strikes of walking or heel tapping (denoted as just “heel strikes” in what follows). From the force data, the onset time of each heel strike and finger tap was obtained. The threshold to determine an onset time was defined as the mean plus 5 times the standard deviation of the noise, and then time intervals for each movement were calculated. The mean and standard deviation of the time intervals were obtained and used to calculate coefficient of variation (CV). To compare mean intervals with the target intervals (Fig. 2A,B) and CVs among the tasks (Fig. 2C,D), a one-way repeated analysis of variance (ANOVA) with a Bonfferoni post hoc criterion was utilized, assuming the homoscedasticity of the data set was acceptable. Otherwise, the Kruskal-Wallis test was utilized.

There were two different types of failure in performance for the dual motor tasks. The first type of failure involved the inability to perform stable finger tapping and/or heel striking when both were done simultaneously. When the subjects performed a single task involving either walking or finger tapping, the CV of the movement was about (or lower than) 5% (Fig. 2A,B). Therefore, we set the threshold for failure at 15% CV. Those subjects who showed CVs greater than the threshold were excluded from the subsequent analysis to evaluate independency of finger and leg movements.

The second type of failure is instability of the relative phase of two movements. To evaluate independency, the relative phase φ between the onset of finger tapping and heel strike was calculated for those subjects who showed stable walking and finger tapping^60–63^. The time interval between two consecutive heel strikes at t_i_ and t_i + 1_ was defined as one cycle (360°), and the relative phase of a finger tapping at the time t_f_ that fell in the period from t_i_ to t_i + 1_ was calculated as φ = 360° × (t_f_ − t_i_)/(t_i+1_ − t_i_). In the self-paced walking, given-paced walking and bipedal heels striking tasks, heel strikes of both legs were gathered as one data set. If a finger tap and a heel strike occurred simultaneously, the relative phase would be 0°.

A histogram was constructed to illustrate distribution of the relative phase for each subject. The entire range of the relative phase (0° to 360°) was divided into 8 bins of 45°. If finger tapping and walking/heel tapping were independent, the probability of an instantaneous relative phase falling in each bin would be p = 1/8. Supposing that the total number of finger taps and heel strikes in combination is M, then the binary distribution for the test of the hypothesis can be expressed as B(M, p). Then, chance levels of the distribution were determined from B(M, p). This was utilized in the evaluation of the number of relative phases in a single bin of the histogram. The number at which the cumulative probability becomes 0.025 was set as the lower limit for the chance level. The number at which the cumulative probability became 0.975 was set as the upper limit of the chance level. Therefore, when a bin of the histogram of a trial had a value above the upper limit or below the lower limit, the null hypothesis that the two movements of the trial were independent was rejected at a significance level of p < 0.05. The ANOVA with Student-Newman-Keuls post-hoc criterion was utilized to compare the numbers of significant bins across the tasks.

A distribution histogram of relative phase was also constructed for each motor task as a group data from those subjects who were not excluded by the criteria of instability (Figs 3 and 4). Eight bins of 45° were also utilized. Finally, the difference among the bins was tested with a one-way ANOVA and a Bonfferoni post hoc criterion.

IBM SPSS Statistics 23 was used for all statistical analyses.
